# Preparative Fractionation of Phenolic Compounds and Isolation of an Enriched Flavonol Fraction from Winemaking Industry By-Products by High-Performance Counter-Current Chromatography

**DOI:** 10.3390/plants12122242

**Published:** 2023-06-07

**Authors:** Ariel Fontana, Andreas Schieber

**Affiliations:** 1 Grupo de Bioquímica Vegetal, Instituto de Biología Agrícola de Mendoza CONICET-UNCuyo, Almirante Brown 500, Chacras de Coria M5528AHB, Argentina; 2 Institute of Nutritional and Food Sciences, Molecular Food Technology, University of Bonn, Friedrich-Hirzebruch-Allee 7, D-53115 Bonn, Germany; schieber@uni-bonn.de

**Keywords:** circular economy, isolation, isorhamnetin, kaempferol, myricetin, polyphenols, purification, quercetin, winemaking by-products

## Abstract

High-performance counter-current chromatography (HPCCC) was used as a tool for the isolation and fractionation of phenolic compounds (PCs) in extracts from wine lees (WL) and grape pomace (GP). The biphasic solvent systems applied for HPCCC separation were *n*-butanol:methyl *tert*-butyl ether:acetonitrile:water (3:1:1:5) with 0.1% trifluoroacetic acid (TFA) and *n*-hexane:ethyl acetate:methanol:water (1:5:1:5). After refining the ethanol:water extracts of GP and WL by-products by ethyl acetate extraction, the latter system yielded an enriched fraction of the minor family of flavonols. Recoveries of 112.9 and 105.9 mg of purified flavonols (myricetin, quercetin, isorhamnetin, and kaempferol) in GP and WL, respectively, from 500 mg of ethyl acetate extract (equivalent to 10 g of by-product) were obtained. The HPCCC fractionation and concentration capabilities were also exploited for the characterization and tentative identification of constitutive PCs by ultra-high performance liquid chromatography-mass spectrometry (UHPLC-MS). In addition to the isolation of the enriched flavonol fraction, a total of 57 PCs in both matrixes were identified, 12 of which were reported for the first time in WL and/or GP. The application of HPCCC to GP and WL extracts may be a powerful approach to isolate large amounts of minor PCs. The composition of the isolated fraction demonstrated quantitative differences in the individual compound composition of GP and WL, supporting the potential exploitation of these matrixes as sources of specific flavonols for technological applications.

## 1. Introduction

Viticulture is one of the most relevant agro-economic activities in the world, with more than 34 million tons of wine grapes produced yearly [1]. The whole winemaking industry generates enormous amounts of by-products [2]. Only in the winemaking process, about 20–25% of the weight of processed grapes is lost as pomace [3]. Thus, around 8 million tons of grape pomace (GP) are produced globally annually, mainly consisting of grape skins and seeds [4]. Wine lees (WL) are the principal by-product obtained after stabilization, initial aging, and filtration of wine. Their main components are yeast and bacteria involved in the winemaking process, tartaric acid salts, precipitated tannins, proteins, inorganic matter, and free phenolic compounds [5,6]. Because of the extremely large amounts of side streams and their susceptibility to spoilage, GP and WL are an ecological and economical issue for the wineries. Therefore, the request for greener industrial production, along with the challenge of minimizing by-product treatment costs, has motivated the search for strategies to utilize these by-products [2]. In the same way, the increase in consumers’ consciousness about the use of additives in food products and the attention that functional ingredients have experienced in recent years has generated a need to identify alternative natural and safer sources of biofunctional and technologically valuable ingredients.

Grape pomace is particularly rich in PCs, mainly anthocyanins and flavanols, that have been extensively characterized [7,8]. Several strategies have been described for the application of crude extracts, isolated fractions, or separated PCs, for example, as natural antioxidants, nutraceuticals, and food preservatives [9]. Although wine lees are the second largest winemaking by-product and contain a wide range of potentially valuable compounds, they have received little attention concerning valorization [10]. Previous research on this matrix showed mostly their richness in anthocyanins, which has been accounted to be up to 10 times higher in concentration than in grape skins/pomace [11]. López–Fernández–Sobrino et al. [12] presented a profiling of PCs in wine lees, reporting 40 anthocyanin derivatives, including many pyranoanthocyanins, flavanols (mainly dimeric and trimeric proanthocyanidins), some flavonols (monomeric and glycosylated derivatives), phenolic acids, and stilbenes. Other authors have also reported qualitative PCs richness in WL, no isolation of the families has been performed, and only a limited number of PCs was identified [11,13,14,15,16,17].

High-speed countercurrent chromatography (HSCCC) is a valuable technique for fractionating families of compounds and/or purifying active constituents from plant extracts prior to the identification of their composition [18,19]. HSCCC is a powerful liquid-liquid partition chromatographic technique without solid support, able to achieve very efficient separation of large sample amounts and high yields [18,20]. The possibility of directly introducing extracts into the separation column without the need for extensive preparation makes it an ideal preparative tool for the isolation and purification of secondary plant metabolites [21,22]. An additional advantage of HSCCC is related to obtaining simplified and concentrated fractions of samples due to the different polarities applied along the run (and the high sample load allowed), helping in the subsequent identification of novel compounds. High-performance CCC (HPCCC) has recently been developed to improve the HSCCC method by combining CCC separation principles with a rapid mixing-separation cycle [23]. HPCCC allows high column rotation speeds (up to 1600 rpm), which achieves approximately four times higher centrifugal force than HSCCC, decreasing separation time without compromising resolution [18]. HSCCC has been applied to fractionate PCs from wine industry-derived matrices such as wines and grape seeds [21,24,25,26,27]. In the case of GP, only two reports apply HSCCC for the isolation of hydroxycinnamoyltartaric acids [28] and anthocyanins [29], where different solvent systems were applied to achieve the purification of these families of PCs. In contrast, no studies on PCs fractionation by HPCCC have been reported for WL. Although most of the previous studies were purified anthocyanins [24,25], proanthocyanidins [21,26], phenolic acids [21], and resveratrol derivatives [27], no attempt has previously been made in the isolation of flavonol constituents from GP and WL. This might be due to the lack of a powerful approach to isolate minor constituents from a mixture composed mostly of major constituents like anthocyanins and flavanols [30]. In this vein, the present work describes a series of processes to demonstrate the suitability of HPCCC for the fractionation and concentration of minor PCs prior to their identification. The isolation of a flavonol-enriched fraction from GP and WL ethyl acetate extracts is also reported. The identification of PCs in the fractions was performed by ultra-high-performance liquid chromatography coupled with mass spectrometry (UHPLC-MS). This is the first time HPCCC has been applied for the fractionation of PCs from GP and WL.

## 2. Results and Discussion

### 2.1. HPCCC Fractionation

HPCCC is an excellent tool for the isolation and purification of bioactive compounds from crude extracts. In comparison with its HSCCC precursor, the application of high column rotation speeds substantially decreases the separation time without compromising the resolution. The successful separation is conditioned on selecting a suitable two-phase solvent system [31]. Although HSCCC has successfully been applied to the isolation and purification of many natural compounds, including flavonoids from various samples [22,30,31,32,33,34], so far, it has not been used for the fractionation, concentration, and isolation of GP and WL PCs.

**Figure 1:**
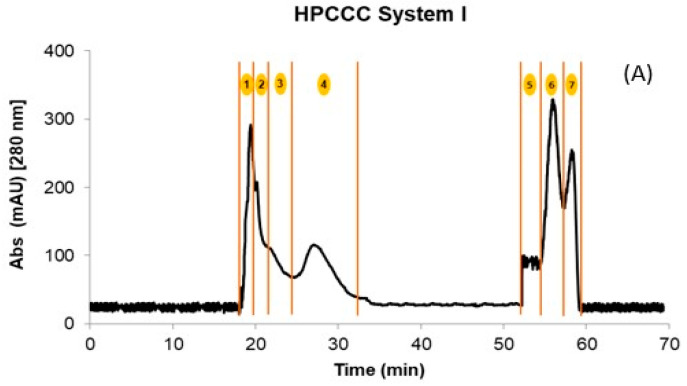

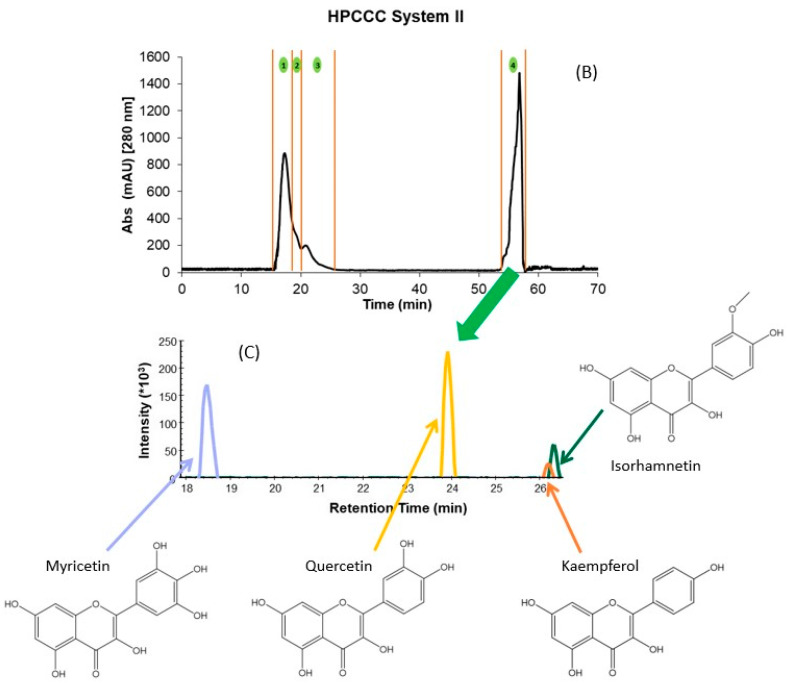
HPCCC fractionation obtained using the different solvent systems. (**A**) System I; (**B**) System II, and (**C**) Overlayed UHPLC-MS extracted chromatograms of the precursor ions for the isolated flavonols and their structures. The numbers 1 to 7 in (**A**,**B**) denote the number of the collected fraction with each of the solvent systems in the specified time range. Skyline 22.2 software was used to process Figure (**C**). Structures were made with ChemDraw 18.2.

Initially, the crude extracts of GP and WL were fractionated by HPCCC using the biphasic solvent system I described in Section 3.3. After 70 min of separation, a total of four fractions in the normal elution mode and three additional fractions in the extrusion mode were obtained for WL. In the case of GP, only two coil fractions were recovered. The quantities obtained in the different fractions after being concentrated and freeze-dried are shown in Table 1. Figure 1A shows a representative HPCCC chromatogram by applying solvent system I for the WL extract. The composition of the fractions was complex, with too many compounds co-eluting in different fractions (Table 2).

For example, independently of the matrix (GP or WL), the flavonols eluted in two fractions at the end of the extrusion mode. In addition, for GP extract, this family also co-eluted with the flavanols procyanidin B1, (+)-catechin, procyanidin B2, and (−)-epicatechin. This hampered the isolation and identification of minor compounds from these matrices due to other PCs at high concentrations (interference effect) (Table 2). Therefore, the crude extracts were re-extracted using ethyl acetate to simplify these initial fractions achieved with system I and concentrating the flavonols. HPCCC was applied to these extracts using solvent system II (Section 3.3), which resulted in a total of four fractions for GP and WL (Figure 1B). The initial sample extract recovery from the two HPCCC solvent systems was 47 and 55% for GP and 45 and 54% for WL in systems I and II, respectively. It should be pointed out that F1 to F4 originating from system I represented on average 88% of total recovered PCs for GP and WL. Based on the identification of compounds by UHPLC-MS, these fractions were rich in anthocyanin derivatives, flavanols, some phenolics acids, and flavonols glycosides (Table 2). For system II, F1 to F3 represented high 62 and 61% for GP and WL, respectively, and the last fraction of extrusion mode (F4) represented 38 and 39% for GP and WL, respectively. In F4 of system II, well-purified fractions of flavonoids were obtained for both matrices. Table 3 presents the calculated amounts of flavonols obtained from HPCCC with both solvent systems in GP and WL. As can be seen, the distribution of compounds was quite different between matrices and systems. WL extract combined similar amounts of myricetin and quercetin when system II was applied, but in GP, quercetin represented 86% of total recovered flavonols. When system I was applied, the flavonols were distributed in the last two extrusion fractions of GP (F5 and F6) and WL (F6 and F7). The flavonols eluted together or in a complex mixture with flavanols like (+)-catechin, (−)-epicatechin, and procyanidin B1 and B2. This co-elution was not observed in system II, where clean chromatograms with myricetin, quercetin, kaempferol, and isorhamnetin as main constituents of F4 were obtained (Figure 1B,C). Flavonols were not detected in the other fractions of system II. Considering the high concentrations found for the compounds, particularly for the less studied myricetin in WL, this matrix can be suggested as a new source of flavanols not explored up to now. Extrapolating this data to a large scale, more than 10 g of this flavonol-rich extract can be obtained from 1 kg of dry by-product. In this context, the results underline the potential of these by-products as a rich source of flavonols like myricetin and quercetin, but also of isorhamnetin. The high amounts of quercetin (86.6%) also suggest an opportunity for its recovery from GP. In fact, due to their different composition, GP and WL can be used for the selective exploitation of different (initially minor) flavonols according to the intended use of the active ingredient.

Considering potential applications, myricetin has been reported to have higher antioxidant activities than quercetin and other flavonoids like naringin, naringenin, rutin, morin, and kaempferol in different lipid systems [35,36]. Flavonols play a role as primary antioxidants by donating a hydrogen atom, acting as free-radical acceptors or chain breakers, and are also able to chelate metals [36]. The higher antioxidant activity can be attributed to the presence of three hydroxy groups at the B-ring. The occurrence of ortho-hydroxyl groups at the 3’ and 4’ positions of ring-B contribute to the improvement of flavonols’ antioxidant activity [36]. Pekkarinen et al. [35] reported that flavonols, especially quercetin, and myricetin, inhibited methyl linoleate oxidation in lipid systems more efficiently than α-tocopherol (the natural antioxidant in oils). The authors also observed that the combination of myricetin or rutin with α-tocopherol exerted a stronger synergistic effect than the use of quercetin. In the same vein, Wanasundara et al. [36] observed that myricetin, (-)-epicatechin, naringin, rutin, morin, and quercetin, were superior to synthetic antioxidants, like butylated hydroxyanisole (BHA) and butylated hydroxytoluene (BHT), in inhibiting canola oil oxidation. Natural flavonoids may therefore have potential applications for the stabilization of oils. The rich composition of GP and WL in the high antioxidant myricetin and quercetin PCs could open a new opportunity for its isolation in high amounts for applications in the food industry due to its high antioxidant power in lipid systems compared to other less antioxidant PCs or synthetic antioxidants. Additionally, the potential application of the whole fraction could be an opportunity due to the potential synergistic effect of flavonols between them and other antioxidants.

### 2.2. Identification of PCs in the Different HPCCC Fractions

The analysis of the different fractions obtained from HPCCC by UHPLC-diode array detection (DAD)-MS allowed the detection of a total of 57 PCs of different families in GP and WL extracts. The identification of PCs in the different fractions was based on acquiring the ESI-MS spectra, in both positive and negative modes. When reference compounds were available, they were confirmed by injecting the pure standard compound. In these cases, in addition to the corresponding MS and UV spectra, the retention time was also employed. Identification of the remaining compounds was based on the mass spectrum (MS/MS fragments) and the bibliographic data collected from previous publications [12,16,37] and the available databases, such as MassBank, PubChem, and ChemSpider.

Table 3 compiles the information related to the PCs found in the different fractions of the HPCCC systems. The number of identified PCs in GP and WL extracts was 36 and 57, respectively. The main difference between both matrices was observed for the anthocyanin composition, mostly in the group of pyranoanthocyanins, for which only two derivatives were found in GP (Table 2, malvidin-3-glucoside-ethyl-epicatechin and malvidin-3-glucoside-vinylguaiacol). The concentration of monomeric anthocyanins constantly declines during wine aging, at the time when complex and stable anthocyanin-derived pigments, like pyranoanthocyanins, are formed. These compounds are anthocyanin-derived pigments formed during the maturation and aging of wine that result from the reaction between anthocyanins or with flavanols or aldehydes [37]. Different mechanisms are involved in the qualitative changes of wine monomeric anthocyanins. Some of them include the absorption of wine components by yeast, their degradation and oxidation, their precipitation with proteins, polysaccharides, or condensed tannins, and the progressive and irreversible formation of more complex and stable anthocyanin-derived pigments [37]. Some of these processes are related to their potential accumulation in WL, as observed here by their detection in this wine industry by-product. The difference between GP and WL anthocyanin composition is related to their different nature. WL are obtained after long contact with the wine, which entails numerous chemical reactions during maturation and aging, also forming new compounds that can accumulate in WL. Regarding the anthocyanins composition of WL, López–Fernández–Sobrino et al. [12] presented a characterization of WL from three varieties, identifying 40 anthocyanins and pyranoanthocyanins. The profile of this family of compounds in the sample analyzed in the present work (Malbec WL) showed a lot of qualitative similarities. Compared to this previous paper and others reporting WL PCs characterization [10,13,14,15,16,17], we tentatively identified pyranoanthocyanins that were not previously found in WL. These included malvidin 3-O-coumaroyl-pyruvic acid, malvidin-6-(caffeoyl)-3-glucoside, delphinidin 3-O-acetylglucoside-piruvic acid, delphinidin 3-O-coumarylglucoside-piruvic acid, delphinidin 3-O-acetylglucoside-4-vinylphenol, peonidin 3-O-glucoside-pyruvic acid, peonidin 3-O-acetylglucoside-4-vinylphenol, petunidin 3-O-glucoside 4-vinylphenol, delphinidin 3-O-glucoside-4-vinylguaicol and peonidin 2-O-acetylglucoside-4-vinylepicatechin (Table 2). These compounds were previously reported in red wines. Their finding in WL agrees with the absorption of PCs in different matrix components, such as residues of yeast/bacteria and proteins. During wine aging, irreversible absorption and interaction of PCs with these components retain significant quantities in WL, making there a good source of bioactive ingredients. Compared with previous studies on WL characterization, the variations may be related to several factors, including the differences in the varieties of grapes and wine elaboration conditions. In addition, their accumulation by HPCCC may be a reason for their detection in the present study. As mentioned in Section 3.1., this concentration of compounds was also observed for other families like flavonols. The HPCCC step with the proper solvent system could be a convenient tool for sample preparation, clean-up, and concentration in chemical profiling studies for new compounds. In addition to the results of previous studies on these derivatives [38], in the present work, two new anthocyanin derivatives were found (malvidin-3-glucoside-ethyl-epicatechin and peonidin 3-O-glucoside-pyruvic acid). These compounds were the only pyranoanthocyanins derivatives found in GP, in comparison with the 21 of this group found in WL fractions. Grape anthocyanins and anthocyanin-derived pigments formed during must fermentation have also been reported in GP [39], but with less diversity probably related to the processes to which it is exposed compared to WL. GP is obtained after the first fermentation of red grapes, and the commonly found anthocyanins result from peel extraction. Normally, not long aging of wine with GP is performed. By analyzing the elution in the HPCCC systems, anthocyanins co-eluted in different fractions (typically the first ones in the normal elution mode) with the most polar solvent phase in both systems. Interestingly, in the case of WL, several pyranoanthocyanin derivatives were found only after the application of HPCCC system II of the ethyl acetate extract. A probable reason for this behavior is related to the concentration effect of this kind of derivative after the extraction with ethyl acetate. Besides the effect of the reduction in the number of fractions of HPCCC achieved with system II, a remarkable result is the qualitative enrichment of PCs. It is interesting that the application of system I on the extracts produced the co-elution of most anthocyanins in more than two fractions (in some cases from 1 to 4, Table 2), which evidenced a lower specificity in the isolation. On the other hand, system II was mostly associated with the elution of this family in WL fractions (generally from 1 to 2). In addition to the less polar composition of the elution phase in this HPCCC system, it could also be related to the previous ethyl acetate extraction. Regardless, these compounds were not isolated in a unique fraction, and their elution with other families like flavanols hamper the potential purification for specific applications.

Considering the flavanols family, 12 derivatives were detected in the different fractions of both HPCCC systems (Table 2). Most of the compounds were found after applying both HPCCC systems and both matrices. Some compounds were found only when system II was used, including procyanidin dimer iso 1, procyanidin dimer iso 2, procyanidin trimer iso 4, procyanidin trimer iso 5, and procyanidin dimer iso 5 in WL and procyanidin dimer iso 2, procyanidin trimer iso 4 and procyanidin trimer iso 5 in GP. This result highlights the relevance of the HPCCC concentration step to allow the tentative identification of minor compounds. The same flavanols presented in Table 2 have previously been reported in WL extracts without HPCCC application [12]. In the case of the GP matrix, previous papers have reported mainly the presence of monomeric and dimeric derivatives. Rockenbach et al. [40] performed a characterization of flavanols in seeds of GP from red varieties identifying, in addition to the monomers (+)-catechin and (−)-epicatechin, several dimers, and trimers as in the present work. Additionally, for flavanols, system II showed simpler elution profiles accounting for most of these compounds in only one HPCCC fraction (F1, WL), compared with system I (commonly distributed in more than 3 fractions, Table 2). The elution of flavanols in system II was achieved in a single fraction, but there were also other PCs in the same fraction as anthocyanins and phenolic acids.

In the case of flavonols, a total of 9 simple and glycosylated derivatives were identified in the fractions. As can be seen from Table 2, the glycosylated derivatives eluted in the initial fractions of both HPCCC systems (F1, S-I and F1-F2, S-II for most of the compounds) together with other PCs like anthocyanins, flavanols, and phenolic acids. For simple flavonols, system I allowed their selective recovery in F6 of GP. In WL with the same HPCCC system, kaempferol and isorhamnetin were isolated in F7 without the co-elution of compounds from other families. Myricetin and quercetin co-eluted between F5-F7 of system I for WL. Except for kaempferol and isorhamnetin in WL, all compounds of this family isolated by HPCCC using system I, independently of the matrix, were also eluting together with other families like flavanols (GP) and some phenolic acids (WL). The application of system II to GP and WL recovered only flavonols in the F4 of the HPCCC extrusion phase in both winemaking by-products. Given the composition of the phases presented in Table 2, the combination of the ethyl acetate extraction of the polyphenols dried extract of by-products and the application of HPCCC system II allowed the isolation of an enriched fraction of flavonols for the first time. The achieved recovery of myricetin in WL for this compound (more than 45% in F4) highlights the potentiality of this wine industry by-product as a source of flavonols. Barcia et al. [16] also reported higher concentrations of myricetin in comparison to quercetin for the WL of different grape varieties. Considering the presented results, the use of HPCCC separation and purification technology may be used for the isolation of minor constituents in extracts of different chemical nature, aiming at the identification and profiling of bioactive compounds (sample preparation focus) and the isolation of an enriched flavonol fraction with focus on its technological application. In terms of the feasibility of the use of HPCCC as an industrial method to obtain these metabolites, evaluation of processes that allows continuous injection of higher volumes of extracts to increase the yield without increasing the solvent consumption and separation time should be investigated for winemaking by-product extracts. This approach has been previously applied to isolate high-purity oleuropein from olive leaf extract [41]. The protocol presented by the authors showed advantages for separating binary mixtures of compounds, such as the large-scale purification of a high-content target compound in the extracts. In our case, some considerations related to the initial concentration of compounds of interest should be taken into account (concentration of a minor fraction of PCs) before the evaluation of this alternative for the industrial production of isolated fractions or individual compounds.

## 3. Materials and Methods

### 3.1. Standards, Solvents, and Materials

Ultrapure water was obtained from a PURELAB Flex 2 water purification system (ELGA LabWater, Paris, France). Ethanol 99% (denatured with benzene, reagent grade) was obtained from Julius Hoesch GmbH (Düren, Germany). Methanol (LC-MS grade), acetonitrile (LC-MS grade), *n*-butanol (99.5%), and trifluoroacetic acid (99%) were purchased from Fisher Scientific GmbH (Schwerte, Germany).

Ethyl acetate (LC-MS grade) and *n*-hexane (97%, HPLC grade) were provided by VWR International GmbH (Darmstadt, Germany). Methyl *tert*-butyl ether (HPLC grade) was purchased from Carl Roth GmbH (Karlsruhe, Germany). Formic acid and the standards of (+)-catechin (≥98%), quercetin (98%) and quercetin-3-O-glucoside were from Sigma-Aldrich (Steinheim, Germany). Malvidin-3-O-glucoside chloride (>97%) was purchased from PhytoPlan GmbH (Heidelberg, Germany). Gallic acid (>98%) was from Fluka (Buchs, Switzerland).

### 3.2. Sample Preparation and PCs Extraction

The WL from Vitis vinifera L. cv. Malbec were obtained as a semisolid residue kindly provided by the Catena Institute of Wine, from Mendoza, Argentina, in the vintage 2021. They were collected after racking the red wine (first-fermentation WL). The WL had been in contact with the wine for three months during alcoholic fermentation, after which WL were isolated and used for this study. The sample was homogenized by agitation at room temperature for 15 min and centrifuged at 855× *g* for 10 min. The solid phase was lyophilized, milled in a mortar, and stored in brown bottles. For the extraction of PCs from Malbec WL, 10 g were extracted with 500 mL ethanol:water acidified with HCl (75:25 *v*/*v*, pH 4). The extraction was carried out for 60 min under continuous stirring at 50 °C. The liquid was filtered through a filter paper and concentrated. A second extraction of the solids was performed with the same conditions. Finally, both extracts were pooled, concentrated utilizing a rotary evaporator at 40 °C, and lyophilized to obtain 2.8 g of dry extract. The extract was resuspended in the HPCCC solvent mixture before analysis or in water in the case of the subsequent liquid-liquid extraction of less polar compounds with ethyl acetate as described subsequently.

The GP sample corresponded to the Cabernet Sauvignon variety and was obtained from 2021 vintage, in Neustadt an der Weinstrasse (Palatinate, Germany). The vinification procedure was conducted with mechanical daily pumping over and contact of the skins and seeds with the juice for 12 days. Subsequently, must was pressed, fresh GP sample was collected and stored at −20 °C until processing. GP sample was ground in a laboratory mixer and lyophilized. The PCs were extracted from 10 g of sample with 500 mL of ethanol:water solution acidified with HCl (50:50 *v*/*v*, pH 2). The solution was incubated for 60 min under continuous stirring at 50 °C. The liquid was filtered through a filter paper and concentrated. A second extraction of the solids was performed with the same conditions. Finally, both extracts were pooled and concentrated using a rotary evaporator at 40 °C. The concentrated extracts were lyophilized to obtain 4 g of dry extract, placed in sealed tubes, and kept in a dry, unlit atmosphere until analysis. Extractions were carried out in triplicate.

To obtain less polar fractions of each matrix, the lyophilized ethanol:water extracts were extracted with ethyl acetate. For this purpose, a portion of 4 g of the GP lyophilized ethanol:water extract was dissolved in 500 mL water and extracted with ethyl acetate (1:1 *v*/*v*) in a separation funnel. The organic phase was removed, and the extraction was repeated twice using 500 mL of ethyl acetate each. The process was carried out in duplicate, and the pooled organic phases were concentrated using a rotary evaporator and lyophilized to obtain 534 mg of dry GP ethyl acetate extract. For WL, the procedure was similar, but 2.8 g of lyophilized ethanol:water extract was used to finally obtain 505 mg of dry WL ethyl acetate extract.

### 3.3. HPCCC Separation

Separations were performed on a Spectrum HPCCC instrument (Dynamic Extractions Ltd., Tredegar, England) coupled with a degasser (model DG1210, Knauer GmbH, Berlin, Germany), an isocratic pump (model 40P, Knauer), a multi-wavelength UV detector (Model 50D, Knauer), a fraction collector (model Foxy R1, Knauer) and a water chiller (model FL1703, Julabo GmbH, Seelbach, Germany) to control the temperature (20 ± 1 °C). The software ClarityChromPrep (Version 5.0.3.192) was used to control all system parameters and data processing. A semipreparative column (136 mL) was used for separation, and different compositions of the solvent system were studied during the separation development. In the case of the extractable PCs extract, the solvent applied to HPCCC consisted of *n*-butanol-methyl *tert*-butyl ether-acetonitrile-water (3:1:1:5) with 0.1%TFA (System I). For the ethyl acetate extract, the following solvent was used: *n*-hexane:ethylacetate:methanol:water (1:5:1:5) (System II). HPCCC was carried out in reverse-phase mode (head-to-tail), where the upper phase (organic) was used as the stationary phase and the lower phase (aqueous) as the mobile phase. The stationary phase was pumped at a flow rate of 10 mL min^−1^ until complete column filling. Subsequently, the rotation speed was set at 1600 rpm, and the mobile phase was pumped into the system at a flow rate of 6 mL min^−1^. When the hydrodynamic equilibrium of the phases was established, 500 mg of the sample prepared as described above was dissolved in 7 mL of the solvent mixture and injected. After an elution time of 28.5 min, the upper phase was used as the mobile phase up to the end of the run (extrusion), and the rotation was kept up to the end of the run. The run was finished after 70 min.

The separation was online monitored at 280 and 520 nm. The fractions were collected in test tubes using the chromatogram obtained at 280 nm and manually combined according to the registered profile. The solvent in fractions corresponding to the peaks resulting from the HPCCC was evaporated employing a rotary evaporator at 40 °C. Finally, the fractions were lyophilized, their yield calculated, and resuspended in the initial mobile phase for analysis by UHPLC-DAD-MS.

### 3.4. Characterization of Fractions by UHPLC-ESI-MS/MS

UHPLC-MS analysis of extracts and fractions was performed on an Acquity UHPLC I-Class system (Waters) consisting of a binary pump, an autosampler cooled at 10 °C, a column oven set at 40 °C, and a DAD scanning from 190 to 800 nm. An Acquity HSS-T3 RP18 column (150 × 2.1 mm; 1.8 μm particle size) combined with a pre-column (Acquity UPLC HSS T3 VanGuard, 100 Å, 2.1 × 5 mm, 1.8 μm), both from Waters, were used for separation. At a flow rate of 0.5 mL/min, analytes were eluted using the following gradient: 0 min, 5% B; 8 min, 10% B; 25 min, 25% B; 26 min, 100% B; 28 min, 100% B; 29 min, 5% B; 31 min, 5% B, with A being water/formic acid (97/3; *v*/*v*) and B being acetonitrile/formic acid (97/3; *v*/*v*). The injection volume was 5 μL. The UHPLC was coupled to an LTQ-XL ion trap mass spectrometer (Thermo Scientific, Inc.) equipped with an electrospray interface (ESI) operating in positive ion mode for analysis of anthocyanins, their derivatives, and flavonols, and in negative ion mode for the other families of PCs. Mass spectra were recorded in the range of m/z 150 to 1500 for identification. Capillary was set at 325 °C with a voltage of 40 V for ESI+ and at 350 °C and a voltage of −44 V for ESI−. The tube lens was adjusted to 70 V for ESI+ and −105 V for ESI−.

## 4. Conclusions

HPCCC was used for the first time for the purification and concentration of PCs in complex matrices such as WL and GP extracts. The proposed application facilitated the fractionation of sample components and, therefore, the characterization and identification of constitutive PCs. The use of an adequate separation system for HPCCC allowed to 1-identify by UHPLC-MS a total of 57 compounds, 12 of which were identified for the first time in WL and/or GP; and 2-obtain a flavonol enriched fraction with high yields in myricetin and quercetin for WL; and mostly quercetin for GP. These results highlight the potential of GP and WL as sources of flavanols with technological applications. The results also suggest that HPCCC may be a convenient alternative to obtain enough amounts of minor PCs, like flavanols from GP and WL, to perform correlation studies between structure, synergy, and bioactivity in food systems.

## Figures and Tables

**Table 1:** Weight measured for the different fractions obtained from the different HPCCC solvent systems in GP and WL.

System I			System II		
Fractions	Weight GP (mg)	Weight WL (mg)	Fractions	Weight GP (mg)	Weight WL (mg)
F1	101	134	F1	150	90
F2	78	45	F2	16	48
F3	55	11	F3	16	27
F4	11	20	F4	113	106
F5	2	2	**Total**	295	271
F6	33	8			
F7	-	15			
**Total**	280	235			

**Table 2:** Identification of the PCs detected in the different fractions obtained using the two HPCCC solvent systems.

PCs	Fraction/s ^a^		Retention Time (min)	[M + H]^+ b^ or [M − H]^− c^	MS/MS Confirmation Fragment
Monomeric Anthocyanins	GP	WL			
delphinidin 3-O-glucoside	F2-F4, S-I	F3-F4, S-I; F1-F3, S-II	7.6	465 ^a^	303
cyanidin 3-O-glucoside	F4, S-I	F4, S-I; F1, S-II	9.6	449 ^a^	287
petunidin 3-O-glucoside	F1-F4, S-I	F1-F4, S-I; F1, S-II	10.9	479 ^a^	317
peonidin 3-O-glucoside	F3-F4, S-I; F1-F2, S-II	F1-F4, S-I; F1, S-II	12.6	463 ^a^	301
malvidin 3-O-glucoside	F1-F4, S-I; F1-F2, S-II	F1-F4, S-I; F1, S-II	13.5	493 ^a^	331
delphinidin 3-O-acetylglucoside	F2, S-I	F1, S-II	14.9	507 ^a^	303
malvidin 3-O-acetylglucoside	F1, S-II	F1-F4, S-I; F1-F2, S-II	20.1	535 ^a^	531
peonidin 3-O-p-coumaroylglucoside	F2, S-I; F1, S-II	F3, S-I; F1-F3, S-II	23.9	609 ^a^	301
Pyranoanthocyanins derivatives					
petunidin 3-O-glucoside-pyruvic acid	n.f.	F2, S-I	12.2	547 ^a^	297
malvidin 3-O-glucoside-pyruvic acid	n.f.	F2-F3, S-I; F1, S-II	15.3	561 ^a^	399
malvidin-3-O-glucoside-acetaldehyde	n.f.	F1-F3, S-II	16.6	517 ^a^	355
malvidin-3-O-glucoside-ethyl-epicatechin	F4, S-I; F1, S-II	F4, S-I; F1, S-II	18.4	809 ^a^	357
malvidin-3-O-acetylglucoside-pyruvic acid	n.f.	F4, S-I; F1, S-II	16.9	603 ^a^	399
malvidin 3-O-coumaroyl-pyruvic acid	n.f.	F1, S-II	20.5	707 ^a^	535
malvidin-6-(caffeoyl)-3-O-glucoside	n.f.	F3-F5, S-I; F1-F2, S-II	21.6	655 ^a^	331
malvidin-3-O-glucoside-vinylcatechol	n.f.	F3-F5, S-I; F1-F3, S-II	21.9	625 ^a^	463
malvidin-3-O-glucoside-vinyl-catechin	n.f.	F1, S-II	18.9	805 ^a^	593
malvidin-3-O-glucoside-vinylguaiacol	F1-F2, S-II	F1-F4, S-I; F1-F3, S-II	23.8	639 ^a^	331
catechin-ethyl-malvidin-3-O-coumaroylglucoside dimer	n.f.	F5, S-I; F1, S-II	23.4	955 ^a^	609
malvidin-3-O-glucoside-acetyl-vinylcatechol	n.f.	F4, S-I; F1-F2, S-II	17.9	667 ^a^	521
catechin-ethyl-malvidin-3-O-coumaroylglucoside dimer	n.f.	F1-F2, S-II	26	955 ^a^	609
delphinidin 3-O-acetylglucoside-piruvic acid	n.f.	F1, S-II	15.9	575 ^a^	273
delphinidin 3-O-coumarylglucoside-piruvic acid	n.f.	F3-F5, S-I; F1-F3, S-II	20.4	679 ^a^	371
delphinidin 3-O-acetylglucoside-4-vinylphenol	n.f.	F3, S-I; F1-F2, S-II	18.7	623 ^a^	419
peonidin 3-O-glucoside-pyruvic acid	F1, S-II	F1-F2, S-II	13.2	531 ^a^	369
peonidin 3-O-acetylglucoside-4-vinylphenol	n.f.	F1, S-II	18.9	621 ^a^	535
petunidin 3-O-glucoside 4-vinylphenol	n.f.	F3, S-I; F1-F3, S-II	21.4	595 ^a^	533
delphinidin 3-O-glucoside-4-vinylguaicol	n.f.	F3-F5, S-I; F1-F2, S-II	19.7	611 ^a^	535
peonidin 2-O-acetylglucoside-4-vinylepicatechin	n.f.	F1, S-II	23	817 ^a^	613
Phenolic acids					
gallic acid	F3-F4, S-I; F1-F3, SII	F1-F3, S-II	1.5	169 ^c^	125
protocatechuic acid	F5-F6, S-I; F2, S-II	F1, S-II	7.3	153 ^c^	109
caftaric acid	F1-F3, S-II	F5, S-I; F1-F3, S-II	3.9	311 ^c^	179
coutaric acid	F3, S-II	F5, S-I; F3, S-II	5.9	295 ^c^	163
caffeic acid	n.f.	F1-F3, S-II	6.3	179 ^c^	135
syringic acid	n.f.	F5-F6, S-I; F3, S-II	9.7	197 ^c^	125
ferulic acid	n.f.	F5, S-I	7.5	193 ^c^	149
Flavanols					
procyanidin B1	F4-F6, S-I; F1-F2, SII	F1-F4, S-I; F1, S-II	4.02	577 ^c^	425
procyanidin dimer iso1	F4, S-I; F1-F2, SII	F1-F4, S-II	4.4	577 ^c^	425
procyanidin dimer iso2	F1, SII	F1, S-II	4.7	577 ^c^	425
catechin	F4-F6, S-I; F1-F3, SII	F1-F4, S-I; F1, S-II	5.2	289 ^c^	245
procyanidin trimer iso2	F4, S-I; F1, SII	F2-F3, S-I; F1, S-II	5.5	865 ^c^	695
procyanidin trimer iso3	F1, SII	F2-F4, S-I; F1, S-II	5.8	865 ^c^	695
procyanidin dimer iso4	F4, S-I; F1-F2, SII	F2, S-I; F1, S-II	6.1	577 ^c^	425
procyanidin B2	F4-F6, S-I; F1-F2, SII	F1, S-I; F1, S-II	7.1	577 ^c^	425
epicatechin	F4-F6, S-I; F1-F3, SII	F1-F4, S-I; F1, S-II	8.66	289 ^c^	245
procyanidin trimer iso4	F1, SII	F1, S-II	9.4	865 ^c^	695
procyanidin trimer iso5	F1, SII	F1, S-II	10.23	865 ^c^	695
procyanidin dimer iso5	F4-F5, S-I; F1-F2, SII	F1, S-II	10.1	577 ^c^	289
Glycosilated flavonols					
quercetin 3-O-galactoside	F2, S-II	F1, S-I; F1-F3, S-II	10.9	465 ^c^	301
quercetin 3-O-glucuronide	F1-F2, SII	F1, S-I; F1-F3, S-II	15.5	477 ^c^	301
quercetin 3-O-glucoside	F1-F2, SII	F1-F2, S-II	15.9	463 ^c^	301
kaempferol 3-O-glucoside	F1, SII	F1-F2, S-II	13.3	449 ^c^	317
isorhamnetin 3-O-glucoside	F4, S-I; F1, SII	F1-F2, S-II	12.7	479 ^c^	316
Simple flavonols					
myricetin	F6, S-I; F4, S-II	F6-F7, S-I; F4, S-II	18.3	319 ^b^	273
quercetin	F6, S-I; F4, S-II	F5-F7, S-I; F4, S-II	23.8	303 ^b^	257
kaempferol	F6, S-I; F4, S-II	F7, S-I; F4, S-II	26.3	287 ^b^	165
isorhamnetin	F6, S-I; F4, S-II	F7, S-I; F4, S-II	26.4	317 ^b^	302

S-I and S-II refer to systems I and system II, respectively. The script (-) between fractions (F) means that the compound was found in all these fractions. ^a^ Fractions are given together con the respective HPCCC solvent systems in which the compound was found. ^b^ [M + H]^+^ was used for the determination of these compounds. ^c^ [M − H]^−^ was used for the determination of these compounds. n.f.: not found.

**Table 3:** Calculated yields in mg and percentages of individual flavonols obtained from HPCCC-specific fractions with different solvent systems in GP and WL extracts. For detailed information about fractions, see Table 1.

	System I				System II			
	GP (mg)	GP (%)	WL (mg)	WL (%)	GP (mg)	GP (%)	WL (mg)	WL (%)
myricetin	2.2	6.6	11.1	37.1	10.7	9.5	48.4	45.7
quercetin	30.1	91.3	17.5	58.4	97.9	86.6	50.6	47.7
kaempferol	0.3	0.9	0.5	1.6	1.6	1.4	3.1	2.9
isorhamnetin	0.4	1.2	0.8	2.8	2.7	2.4	3.8	3.6

Results are expressed as mg of each individual compound concerning the total weight of the fraction in which the flavonols family was determined in each HPCCC system and matrix. The % values were calculated as the proportion of each individual compound regarding the total sum of flavonols (which was considered 100%).

## Data Availability

The data presented in this study are available in Table 1, Table 2 and Table 3, and Figure 1. For additional data, the data will be available by contacting the authors.
